# A Substitute for Vulcanite

**Published:** 1870-04

**Authors:** J. H. Warner

**Affiliations:** St. Clair, Mich.


### Correspondence.

### A SUBSTITUTE FOR VULCANITE.

St. Clair, Mich., Jan. 28, 1870.

Editor Dental Register.—*Dear Sir*:—Knowing that anything likely to serve as a substitute for vulcanite will be hailed with pleasure by the profession, I write to give you my experience with Weston's metal.

Having failed to obtain anything like a fair settlement with Josiah's brigands, I of course was invited by the authorities to quit infringing on the company's rights. I am an amiable man, and accepted the invitation. Previously I had investigated the merits of Rose Pearl, and though sorely against my inclination, I was convinced that as yet, this was not the desired base, as it was expensive, and far more difficult of manipulation than vulcanite, so much so that this feature alone rendered its unpopularity almost certain. Then I was led to try Dr. Weston's metal by a gentleman who resides in the town of Towanda, Penn., and who spoke of the Doctors success at home with his metal in such candid, but complimentary terms, that I was led to a partial belief in its merits.

Determined to try all things, and even abandon plate work before succumbing to the Vulcanite Company, I began the use of Weston's metal, and though my experience has been short, I am so thoroughly convinced that it is equal to vulcanite, that I would not give $5.00 for a company's license to day. Many of my patients who have had temporary plates substituted with Weston's metal give a decided preference to the latter as being more cleanly, and even more nearly tasteless than vulcanite—produces less inflammation of the mucous membrane, and is not sufficiently heavy to be objectionable, even for full upper plates, while for lower plates its weight is a decided advantage, and it is, perhaps, twice or three times as strong as vulcanite.

In addition, it is free from the leprosy of a license, and will prove in the end, I am confident, the trump that will *euchre* the vulcanite company.

If this brief letter should have the effect to cause one member of the profession to abandon vulcanite, its object will have been accomplished.

Truly yours, J. H. WARNER.

